# Hesitancy towards a COVID-19 vaccine among midwives in Turkey during the COVID-19 pandemic: A cross-sectional web-based survey

**DOI:** 10.18332/ejm/143874

**Published:** 2022-01-26

**Authors:** Leyla Kaya, Yasemin Aydın-Kartal

**Affiliations:** 1 Zeynep Kamil Women and Children's Diseases Training and Research Hospital, Gynecology Clinic, Istanbul, Turkey; 2 Department of Midwifery, Faculty of Health Sciences, University of Health Sciences, Istanbul, Turkey

**Keywords:** vaccine acceptance, COVID-19, vaccine hesitancy, midwives

## Abstract

**INTRODUCTION:**

One of the most significant barriers to social immunization, which is critical in combating the COVID-19 pandemic, is vaccine hesitancy or rejection. The purpose of this study was to determine the acceptance, hesitancy and barriers to COVID-19 vaccines among midwives in Turkey.

**METHODS:**

A total of 806 midwives participated in the cross-sectional study, which was conducted online from November 2020 to January 2021. The data were collected by using an Introductory Information Form, Anti-Vaccination Scale - Short Form, and Attitudes to the COVID-19 Vaccine Scale.

**RESULTS:**

In all, 17.2% of the midwives in the study had a history of COVID-19 infection, which was confirmed by a PCR test; 69% were exposed to COVID-19 patients; 36.8% had a person diagnosed with COVID-19 with PCR in their family; and 18.1% had a relative die due to COVID-19. In the study, 16.8% of midwives considered getting the COVID-19 vaccine, while the majority (48.8%) stated they would get the vaccine once vaccine safety was established, while 10.5% stated that they did not wish to receive the vaccine. Insufficient phase studies of COVID-19 vaccine studies (75.6%) and insufficient control due to imported COVID-19 vaccines developed (48.1%) were among the most important determinants of COVID-19 vaccine reluctance.

**CONCLUSIONS:**

The potential acceptance rate of COVID-19 vaccines by the study midwives was found to be low. The knowledge, confidence and attitude of midwives toward vaccines are important determinants of patients’ vaccine acceptance and recommendation.

## INTRODUCTION

As of 13 January 2021, COVID-19 infection had spread to 219 countries, causing the death of about 2 million people^1^. As of 13 December 2020, the total number of infected people reported by the Ministry of Health since the beginning of the pandemic in Turkey is over 1.8 million and the number of deaths is 16417^2^. The pandemic caused by a new coronavirus, SARS-CoV-2, is the most significant public health problem of the 21st century. The high contagion, its unprecedented negative impact on countries’ health systems, and the lack of treatments that can improve the disease’s prognosis to date highlight the importance of developing an effective and reliable vaccine against this disease. Furthermore, it is suggested that vaccines are the only way to achieve community immunity required to end the pandemic^3^.

One of the most significant barriers to social immunization, which is critical in combating the COVID-19 pandemic, is vaccine hesitancy or rejection. Understanding the dynamics of vaccine trust has always been critical for public health. There are numerous reasons why anti-vaccination sentiment has resurfaced in the midst of the COVID-19 pandemic. These reasons could include conspiracy theories as well as concerns about the vaccine’s manufacturing process and utility^4^.

Medical personnel are at high risk of contracting viruses such as influenza and SARS-CoV-2^5^. A study revealed that healthcare professionals (27%) are as hesitant to get vaccinated as the general population (29%)^6^. The reasons for health personnel’s concerns about the vaccine have been reported as an insufficiency of vaccine information and an unknown potential long-term side effect^7^. Healthcare workers who are role models for society have important implications for creating attitude and behavior change^8^. A successful immunization program with high health worker participation will reduce direct and indirect costs by ensuring the continuation of healthcare during epidemics and pandemics, as well as immunization of health workers^8,9^. Therefore, it is critical to examine COVID-19 vaccine hesitancy and vaccine acceptance dynamics in populations planned to receive the first vaccine, such as healthcare workers and vaccine prescribers. In addition, COVID-19 immunization did not begin in Turkey at the time of the study’s application, and the COVID-19 vaccination program began on 14 January 2021, with priority medical personnel.

## METHODS

This cross-sectional study was conducted between 15 December 2020 and 10 January 2021. This study was conducted to determine the potential acceptance of COVID-19 vaccines among Turkish midwives during the COVID-19 pandemic, vaccine hesitancy, and factors influencing vaccine acceptance.

### Study design, setting and sample

The population of the research consisted of about 2600 midwives who are members of the Anatolian Midwives Association and about 3750 midwives who are members of the Turkish Midwives Association, two major professional associations in Turkey. There was no sample selection in the study, and 806 midwives who were active and volunteered to participate in the study formed the study’s sample. The study survey was distributed to member midwives via various social media platforms, e-mail, or messaging apps, by provincial association representatives using the https://www.google.com/forms/about/ web address.

### Data collection forms

An Introductory Information Form, an Anti-Vaccination Scale - Short Form, and an Attitudes to the COVID-19 Vaccine Scale were applied to midwives who agreed to participate in the study. All participants were informed about the study’s purpose online, and they were invited to participate. Because the survey posed only a minor risk to the subjects and did not include any procedures that would normally necessitate written consent outside of the study context, online approval was obtained. No identifying information was added to the online survey to ensure the confidentiality of participant information.

### Introductory information form

This was organized by researchers who conducted literature research and consulted experts. Midwives’ sociodemographic and professional characteristics (age, education level, family type, income level, duration of clinical studied professional experience, etc., see Supplementary file) and information about COVID-19 infected patients, if healthcare workers themselves were infected from COVID-19 exposure, vaccine-related beliefs and attitudes, and vaccine acceptance, were included on the form.

### Anti-vaccination scale - short form (AVS)

The scale was created in a 5-point Likert scale style by Kılınçarslan et al.^10^ to determine vaccination hesitancy in individuals over the age of 18 years. There is no calculated cut-off value for a 12-item short form. Vaccine opposition/ hesitancy increases as the score rises. There are three sub-dimensions of the scale: ‘Vaccine Benefit and Protective Value’, ‘Anti-Vaccination’ and ‘Solutions For Not Being Vaccinated’. The Cronbach Alpha value of the scale is 0.85. The Cronbach Alpha value for this study was 0.82.

### Attitudes to the COVID-19 vaccine scale (AVS-COVID-19)

The scale developed by Geniş et al.^11^ consists of 9 items and two sub-dimensions (positive and negative attitudes). In the lower dimensions of negative attitude, items are scored in reverse. High scores from the positive attitude sub-dimension indicate an attitude towards vaccination, while high scores from the negative attitude sub-dimension indicate a less negative attitude towards vaccination. The Cronbach Alpha value of the scale is 0.74. The Cronbach Alpha value for this study was 0.76.

### Statistical analysis

The data were analyzed on SPSS (Statistical Package for Social Sciences) 22.0 software. Descriptive statistical methods such as frequency, percentage, mean, SD, and the Kolmogorov–Smirnov distribution test for normal distribution were employed during the data analysis. Reliability was assessed using Cronbach’s alpha test. In accordance with the nonparametric methods, Mann–Whitney U test (Z Table value) was used for comparing means of two independent groups and a Kruskal–Wallis H (χ2 Table value) test to compare means of three or more independent groups. The statistical results were considered significant at the level of p<0.05.

## RESULTS

**Figure 1:**
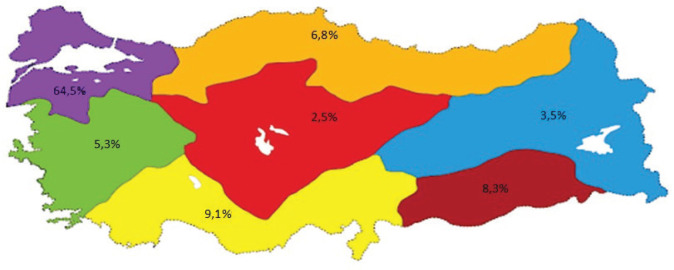
Regional distribution of participants (n=806)

Midwives active in the health sector were invited to the study and 806 midwives agreed to participate. The average age of participants was 32.65 ± 8.24 years, they were mostly aged 20–39 years (80.4%) and they preferred the midwifery profession willingly (71.2%). In all, 64.5% of participants were from the Marmara Region, 9.1% from the Mediterranean Region, 8.3% from the Southeastern Anatolia Region, 6.8% from the Black Sea Region, 5.3% from the Aegean Region, 3.5% from the Eastern Anatolia Region and 2.5% from the Central Anatolia Region (Figure 1).

The majority of the midwives participating in the research worked in public hospitals (54%), they mostly worked in COVID-19 clinics (19.5%), delivery rooms (17%), primary care clinics (14.3%), gynecology clinics (10.9%), child clinics (8.9%), mixed clinics (7.6%), emergency services (7.1%), neonatal intensive care (6.3%), intensive care (4.5%), and women’s health and diseases polyclinics (4%).

Of the participants, 17.2% reported a history of COVID-19 infection confirmed by a polymerase chain reaction (PCR) test; 69% were exposed to COVID-19 patients, while 36.8% had a person diagnosed with COVID-19 with PCR in their family, and 18.1% had a relative die due to COVID-19; 36.4% found the measures taken for COVID-19 inadequate, while 23.8% did not believe the pandemic would end with a return to normal life (Table 1).

**Table 1:** Participants’ attitudes and experiences with COVID-19 infection, Turkey, December 2020 to January 2021 (N=806)

*Attitudes and Experiences*	*n*	*%*
**Diagnosed with COVID-19**		
Yes	139	17.2
No	667	82.8
**Having a family members diagnosed with COVID-19**		
Yes	297	36.8
No	509	63.2
**Providing care to patients diagnosed with COVID-19**		
Yes	556	69.0
No	250	31.0
**Death of a family members due to COVID-19 disease**		
Yes	146	18.1
No	660	81.9
**COVID-19 news follow-up frequency** (hours)		
0–0.5	368	45.7
>0.5 to 1	253	31.4
>1 to 2	116	14.4
>2 to 4	50	6.2
>4	19	2.4
COVID-19 news tracking tools*		
Internet	747	92.7
Social media	616	76.4
TV	571	70.8
Health workers	523	64.9
Close friends	329	40.8
Newspaper	85	10.5
Scientific research and authority views	9	1.1
**Do you find the measures taken for COVID-19 sufficient?**		
Insufficient	293	36.4
Partially sufficient	411	51.0
Enough	85	10.5
Quite sufficient	17	2.1
**Do you believe that pandemic will end with a return to the previous life routine**		
Yes	249	30.9
No	192	23.8
Partially	365	45.3

* More than one answer was given.

Of midwives in the study, 16.8% considered getting the COVID-19 vaccine, while the majority (48.8%) stated they would get the vaccine once vaccine safety was established; 10.5% stated that they did not wish to receive the vaccine (Table 2).

**Table 2:** Participants’ opinions about COVID-19 vaccines, Turkey, December 2020 to January 2021 (N=806)

*Vaccination Status and Opinions*	*n*	*%*
**COVID-19 vaccination status**		
Yes, I want to be vaccinated, as soon as possible.	135	16.8
No, I will postpone vaccination until vaccine safety is approved.	393	48.8
No, I don’t ever think of getting it done.	85	10.5
I am indecisive.	193	23.9
**Did you take the influenza vaccine during the last year?**		
Yes	62	7.7
No	744	92.3
**Media reports cause hesitancy about COVID-19 vaccine**		
Yes	647	80.3
No	159	19.7
**COVID-19 vaccination for your relative who is aged ≥65 years with chronic disease**		
Yes	380	47.1
No	426	52.9
**Will vaccines developed for COVID-19 be effective in ending the pandemic?**		
Yes	304	37.7
No	502	62.3

The vast majority of participants (80.3%) believed that news in the media creates skepticism about the COVID-19 vaccine, and 62.3% did not believe that COVID-19 vaccines will be effective in ending the pandemic (Table 2).

When evaluating the reasons why midwives in the study did not receive COVID-19 vaccines, the most common reasons were identified as: insufficient clinical phase trials of COVID-19 vaccines (75.6%), insufficient inspection due to imported COVID-19 vaccines developed (48.1%), and not believing COVID-19 vaccines are safe (41.8%) (Table 3).

**Table 3:** Reasons for unwillingness to receive COVID-19 vaccines, Turkey, December 2020 to January 2021 (N=806)

Vaccination reluctance reasons*	*n*	*%*
I think the clinical phase studies of the COVID-19 vaccine are insufficient.	610	75.6
Adequate inspection is not passed due to the import of COVID-19 vaccine.	388	48.1
I don’t think the COVID-19 vaccine is safe.	337	41.8
I don’t think the COVID-19 vaccine is beneficial.	316	39.2
Negative news in the press affects my decision.	288	35.7
I don’t believe that the effectiveness of the COVID-19 vaccine will decrease because the virus mutates.	372	33.7
I don’t want to be vaccinated because I find the information about the virus outbreak insufficient.	192	25.8
I don’t find the protection of COVID-19 vaccine sufficient.	170	21.1
COVID-19 vaccine, infertility, autism, cerebral palsy, etc. I think it causes diseases.	145	18.0
I believe the cold chain was broken at customs while vaccines were brought in.	126	15.6
I do not have enough information about the COVID-19 vaccine.	89	11.0
The presence of harmful substances such as mercury and aluminum in the content of vaccines.	79	9.8
I think that vaccines contain substances (pig products, etc.) that are objectionable to my religious beliefs.	37	4.6

* More than one answer was given.

The status of midwives such as age, having children, having had COVID-19 infection, and having contact with COVID-19 positive patients, were found to significantly differentiate midwives’ attitudes toward the COVID-19 vaccine. It was determined that COVID-19 infection status significantly affected the Anti-Vaccination and Solutions For Not Being Vaccinated sub-dimensions average scores. It was also determined that the regions where midwives lived differed in the overall scores of the anti-vaccination scale (Table 4).

**Table 4:** Comparison of the mean scores of the total and sub-dimensions of the anti-vaccination scale (AVS) and the attitudes to the COVID-19 vaccine scale according to sociodemographic and some characteristics of midwives, Turkey, December 2020 to January 2021 (N=806)

		*AVS – vaccine benefit and protective value dimension*	*AVS – anti-vaccination dimension*	*AVS – solutions for not being vaccinated sub-dimensions*	*AVS – total score*	*AVS – COVID-19 positive attitude*	*AVS – COVID-19 negative attitude*
		*mean±SD*	*mean±SD*	*mean±SD*	*mean±SD*	*mean±SD*	*mean±SD*
**Age** (years)	20–29 (n=331)	10.45±3.74	14.80±4.39	7.19±2.52	32.45±8.20	11.44±4.29	14.79±4.61
	30–39 (n=317)	10.30±4.14	14.86±4.49	7.30±2.65	32.48±9.18	11.91±4.45	14.08±3.88
	40–49 (n=93)	9.75±4.06	14.88±4.62	7.04±2.61	31.67±9.79	12.09±4.05	13.74±4.55
	50–59 (n=65)	9.46±3.51	13.29±4.73	6.49±2.45	29.24±8.29	12.40±3.42	13.36±3.47
	p	0.082	0.217	0.180	0.104	0.026 d>a	0.003 a>c, a>d
**Institution**	Public hospital (n=435)	10.23±3.99	14.60±4.65	7.13±2.66	31.96±8.67	11.60±3.95	13.75±4.05
	Private hospital (n=141)	9.65±3.18	14.35±4.08	7.04±2.42	31.04±7.56	12.12±4.33	14.20±4.47
	Primary care clinics (n=149)	10.82±4.43	15.20±4.57	7.08±2.61	33.11±10.51	12.46±4.58	13.65±4.85
	University hospital (n=81)	10.19±3.65	15.08±4.22	7.69±2.31	32.97±8.14	11.95±4.50	13.56±4.46
	p	0.303	0.138	0.803	0.208	0.341	0.378
**Having children**	Yes (n=559)	10.14±3.65	14.51±4.44	7.12±2.50	31.78±8.35	12.10±4.06	12.10±4.17
	No (n=247)	10.44±4.49	15.16±4.62	7.26±2.75	32.87±9.78	11.56±4.56	11.56±4.67
	p	0.934	0.068	0.447	0.253	0.022	0.003
	Yes (n=139)	10.57±3.95	15.47±4.36	7.51±2.52	33.55±9.06	11.61±4.15	14.49±4.36
**Diagnosed with COVID-19**	No (n=667)	10.04±3.90	14.27±4.52	6.96±2.59	31.28±8.58	12.11±4.26	13.55±4.30
	p	0.076	0.000	0.000	0.000	0.085	0.002
**Having a family member diagnosed with COVID-19**	Yes (n=297)	10.28±3.95	14.62±4.31	6.97±2.63	31.87±7.89	11.94±3.98	13.75±4.15
	No (n=509)	10.22±3.92	14.73±4.54	7.20±2.57	32.16±9.01	11.93±4.27	13.93±4.38
	p	0.896	0.852	0.197	0.687	0.882	0.409
**Providing care to patients diagnosed with COVID-19**	Yes (n=556)	10.38±4.07	14.87±4.58	7.25±2.65	32.51±9.23	12.46±3.84	14.03±4.46
	No (n=250)	9.91±3.58	14.36±4.31	6.95±2.41	31.22±7.80	11.69±4.37	13.61±4.06
	p	0.328	0.078	0.084	0.051	0.003	0.128
**Regional distribution of participants**	Marmara Region (n=519)	10.48±3.86	13.76±4.79	6.34±2.45	29.20±9.05	11.82±4.09	14.11±4.16
	Aegean Region (n=43)	10.21±3.90	13.35±5.12	6.14±1.89	29.71±8.38	12.13±4.76	13.41±4.72
	Black Sea Region (n=55)	9.65±4.72	14.45±4.63	6.94±2.20	30.10±8.01	13.23±3.74	13.41±4.73
	Central Anatolia Region (n=20)	9.38±3.64	14.96±4.72	7.22±3.15	31.58±8.67	12.15±5.67	13.45±4.43
	Eastern Anatolia Region (n=28)	10.62±4.80	14.95±4.41	7.28±2.57	32.73±8.94	12.70±4.83	13.16±4.92
	Southeastern Anatolia Region (n=67)	9.70±3.54	14.11±4.93	7.13±2.69	31.88±7.78	11.22±4.55	13.35±5.02
	Mediterranean Region (n=74)	9.09±3.95	13.15±3.26	6.65±3.03	29.45±9.40	12.14±3.86	13.42±4.08
	p	0.510	0.185	0.068	**0.044 e>a**	0.350	0.512

It was found that the institution in which participating midwives worked and the presence of relatives who had COVID-19 infections in the family did not differentiate the attitudes of midwives towards COVID-19 vaccines, while these variables also did not significantly affect the average score of the Anti-Vaccination Scale (Table 4).

## DISCUSSION

Developing an effective and safe vaccine is a critical step in the struggle against the SARS-CoV-2 virus, which is considered a pandemic and has infected millions of people. The potential vaccination rate of midwives, key members of the medical team, was evaluated in this study, and it was discovered that only 16.8% considered getting a COVID-19 vaccine. The majority of participants (48.8%) stated that if vaccine safety were to be achieved, they would receive the COVID-19 vaccine, while 23.9% were undecided. Clinical trial stages can be shortened or accelerated in unusual cases where there is a need for rapid treatment options, such as the COVID-19 pandemic, to allow for the vaccine’s release. As a result, this may cause people to hesitate to get vaccinated. In fact, the lack of clinical development phase studies (75.6%) and concerns about vaccine safety were the main reasons why midwives participating in the study did not want to receive the COVID-19 vaccine (41.8%).

One of the major reasons for vaccination reluctance among midwives was the importation of the COVID-19 vaccine (48.1%). COVID-19 vaccine development studies are being conducted in 14 different centers across the country^12^. This implies that national vaccines developed in Turkey cannot yet be used for immunization, requiring the use of imported vaccines^13,14^. The fact that vaccines are imported supports the notion that midwives’ effectiveness and safety tests are insufficient.

Negative press coverage of the COVID-19 vaccine (35.7%) is another major source of vaccine apprehension. Many arguments about the scientific existence of the vaccine have been made in the written and visual press since the pandemic^15^. Media platforms (including social media) have also been extremely effective in spreading vaccine hesitations^5^. Unfounded media reports claiming that vaccines contain objectionable substances such as pork products (4.6%) according to the Islamic faith or hazardous substances (mercury, aluminum, etc.) (9.8%), that the COVID-19 vaccine causes infertility, autism, cerebral palsy, and other diseases (18%), and that the COVID-19 virus is a laboratory-produced virus (19%)^16,17^, have had a detrimental impact on attitudes towards COVID-19 vaccines. Social media platforms provide opportunities not only for the anti-vaccination movement, but also for the public health movement. As a result, research to increase public confidence in the vaccine must be carried out by effective people in public opinion who contact the Ministry of Health, use social media, technology, and other media communication tools, and enlighten the public with scientific data. When midwives’ attitudes toward the COVID-19 vaccine were assessed using sociodemographic factors, it was discovered that the positive attitude toward the vaccine increased with participant age. The contamination risk of COVID-19 virus is the same for everyone, but it is also known that its lethal effect increases with age^18^, particularly those over the age of 60 years^19^ and individuals with serious chronic medical conditions who are at higher risk^20^. The severe occurrence of COVID-19 in old age and those with comorbidity^21^ explains the positive vaccination attitude of midwives aged 50–59 years.

The attitudes of midwives who had children towards the COVID-19 vaccine were found to be significantly higher. Similarly, in the study of Uyar et al.^22^, it was reported that the attitudes and behaviors of participants who had children related to vaccines and vaccination were more positive. It can be explained by the fact that those who have children are more concerned about vaccination because they feel responsible for others in addition to themselves and protect their families.

Midwives who had COVID-19 infection had significantly lower negative attitudes towards the COVID-19 vaccine, while those midwives had significantly lower attitudes towards the vaccine. In addition, midwives who had contact with COVID-19 positive patients had a significantly higher positive attitude to the COVID-19 vaccine. In a Siren study conducted in 20787 health workers in the UK, 6614 medical personnel who had COVID-19 and 14173 negative cohort groups were followed up with regular antibody and PCR tests, and 44 patients who had COVID-19 developed re-infection and 409 new infections in the negative cohort group. In the first 5 months after infection, protection lasts 83%, but in this process, infection in medical personnel can continue and the possibility of re-infection has been noted^23^. Antibody levels have been found to be present 5–6 months after infection, according to studies. As a result, vaccination against re-infection after infection is recommended at the earliest three months and no later than six months, accompanied by available evidence^23,24^. Because of the high likelihood of re-infection, it is assumed that at-risk midwives have a positive attitude to COVID-19 vaccines.

When the anti-vaccine attitude of midwives was assessed according to the regions in which they lived, it was observed that the anti-vaccine/hesitancy was highest in the Eastern Anatolia Region and lowest in the Marmara Region. In Turkey, there were approximately 23000 vaccine centers in 2017, with more centers in the Eastern and Southeastern Anatolia region than in other regions^25^. Vaccination rates in Turkey were the highest in the West (Marmara and Aegean Regions), according to Turkish Population and Health Research data from 2018^26^. Conducting descriptive studies of regions rejecting the vaccine will be extremely valuable in identifying the source of the problem and developing solutions.

### Limitations

This study has several limitations. First, because reaching individuals was impossible due to the COVID-19 pandemic, the easy sampling method was chosen as a sampling method. Despite the fact that data from midwives working in all regions of Turkey have been collected, data cannot be generalized to the entire population due to the simple sampling method. Due to limitations in the face-to-face execution of the study during the current active COVID-19 pandemic, the research was conducted using an online survey.

The limitations of cross-sectional polling, such as sampling, response, and recall biases, apply to this study. Finally, the study was carried out at a time when potential COVID-19 vaccines, which can affect information levels, perceptions, and attitudes, were heavily publicized. Despite these limitations, the study emphasized the importance of addressing midwives’ perceptions and attitudes toward potential COVID-19 vaccines, as well as providing information from credible sources to contribute to better vaccine acceptance by health workers.

## CONCLUSIONS

The potential acceptance rate of COVID-19 vaccines by midwives was found to be low. Inadequate clinical phase studies of COVID-19 vaccines, the state of vaccine importation, and vaccine safety concerns have led to vaccination hesitancy among Turkish midwives. Negative attitudes toward vaccines, as well as uncertainty or reluctance to vaccinate, are the primary barriers to long-term COVID-19 epidemic management. The community should create awareness campaigns to correct false information about vaccines and create trust and demand for them by people for their own health. This will ensure the safety and effectiveness of the vaccine and also provide transparent information about the technology used in vaccine production.

## Data Availability

The data supporting this research are available from the authors on reasonable request.
